# Stimulated Emission Tomography of Spontaneous Four-Wave Mixing in Plasmonic Nanoantennas

**DOI:** 10.1021/acsphotonics.5c00764

**Published:** 2025-07-18

**Authors:** John Yang, Xiaofei Xiao, Jefferson Flórez, Nathan Gemmell, Paul Dichtl, Sylvain D. Gennaro, Stefan A. Maier, Chris C. Phillips, Alex S. Clark, Rupert F. Oulton

**Affiliations:** † The Blackett Laboratory, Department of Physics, 4615 Imperial College London, London SW7 2BW, U.K.; ‡ School of Physics and Astronomy, Monash University, Clayton, Victoria 3800, Australia; § Department of Electrical and Electronic Engineering, University of Bristol, Bristol BS8 1FD, U.K.

**Keywords:** stimulated emission tomography, spontaneous four-wave mixing, four-wave mixing, plasmonics, nonlinear optics, photon-pair generation.

## Abstract

We used stimulated emission tomography (SET) to assess the efficiency of spontaneous four-wave mixing (SFWM) from a plasmonic nanoantenna under pulsed excitation. We characterize the SFWM photon generation rate by measuring stimulated degenerate four-wave mixing. We produce a map of the SFWM joint spectral density that characterizes the biphoton state, which we find has a broad bandwidth due to the absence of phase matching. The joint spectral density retrieval via SET is fast and straightforward compared to traditional coincidence measurements. By calculating the number of stimulating and generated photons along with the frequency mixing efficiency, we have determined the power-independent intrinsic SFWM generation rate to be on the order of 10^3^ photon pairs per second per mW squared per particle, while the power-independent extrinsic generation rate is approximately 1 photon pair per second per mW squared per particle. There is scope to increase the nonlinear response by scaling up to large area metasurfaces using low-loss dielectric materials that would allow the produced photon pairs to exceed background fluorescence. Such an SFWM metasurface could be a potential alternative to parametric down conversion, which requires rarer second-order nonlinear materials that are also challenging to integrate with photonic structures.

## Graphical abstract

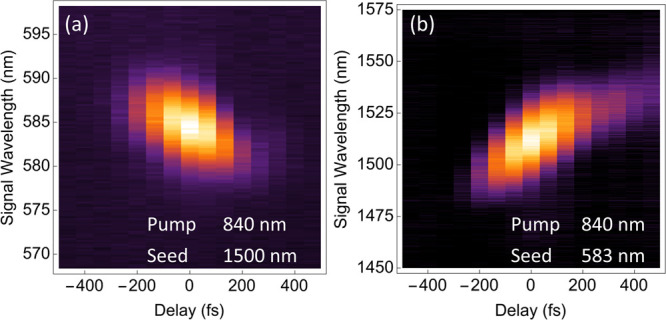

## Introduction

Plasmonic nanoantennas and metasurfaces are extensively used for nonlinear frequency mixing due to their strong electromagnetic field enhancement and femtosecond-scale time response.^1–6^ Similar nonlinearity has been demonstrated in dielectric structures,^7–9^ and while dielectric antennas generally possess a higher damage threshold, plasmonic antennas have the advantages of smaller feature sizes and thus stronger field enhancements.^4,10,11^ By engineering nanoantenna resonances and geometries, researchers have demonstrated effective control over a variety of nonlinear processes.^4^

Recently, there has been interest in using thin nonlinear materials for spontaneous parametric downconversion (SPDC) in order to generate correlated photon pairs for quantum optics applications. This has recently been demonstrated in nanoantennas,^12^ metasurfaces,^13,14^ and thin films,^15^ with SPDC efficiencies reported to be on the order of 10^–2^ pairs per second per mW.^4,14,15^ An alternative method to SPDC is spontaneous four-wave mixing (SFWM), which has not received the same level of attention since it is a weaker phenomenon. SFWM has been studied in films of 100 nm diameter carbon nanotubes^16^ and half micron thick SiN,^17^ but not yet in metasurfaces to our knowledge, which thus warrants investigation. There are several differences that make SFWM an interesting alternative for photon-pair sources. First, it is a third-order nonlinear process, which allows a wider range of candidate materials to be considered, both crystalline and amorphous, since second-order materials rely on broken centrosymmetry. Second, nanoscale nonlinear optics in thin films or arrays of subwavelength-sized structures usually requires intense ultrafast pulses, but SPDC, being a process linearly dependent on pump intensity, cannot use this feature to boost average photon generation rates. It remains to be seen whether SFWM in nanoscale materials can take advantage of the quadratic scaling with pump power being available. Finally, SFWM has the potential for a broader separation of signal/idler wavelengths due to it producing daughter photons at both higher and lower energies than the pump (Figure 1a,b). SPDC, on the other hand, produces downconverted daughter photons that are both at lower energies than the pump. Naturally, the following question arises: What is the generation rate of SFWM photon pairs, and would a thin nonlinear down-conversion source be viable?

**Figure 1:**
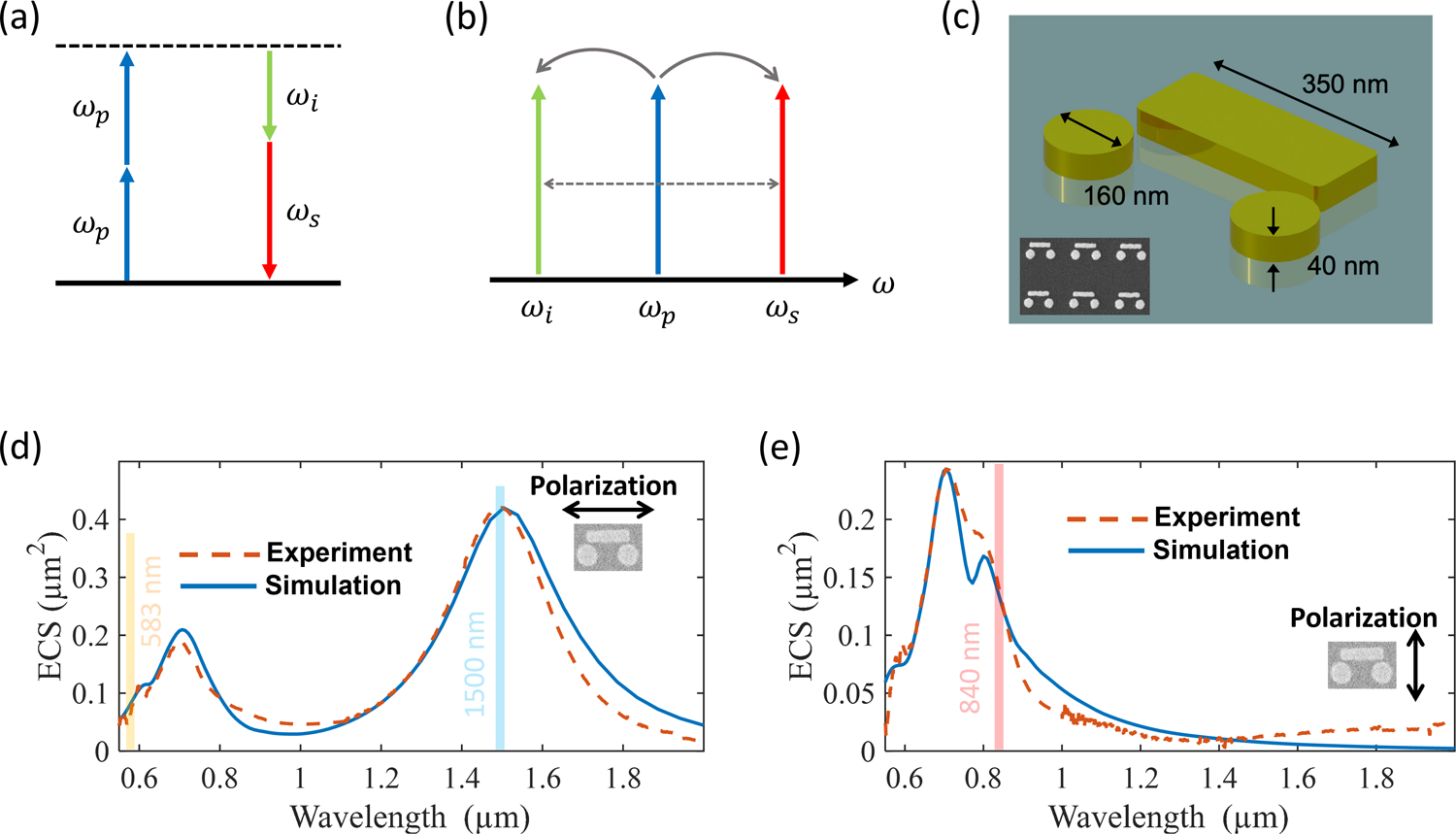
Illustration of four-wave mixing using multiresonant gold nanoantennas. (a) Energy diagram for four-wave mixing. Two pump photons at a frequency ω_p_ are annihilated to produce an idler photon with frequency ω_i_ and signal photon with frequency ω_s_. (b) In four-wave mixing, the signal/idler photons have higher/lower energies than the pump. (c) Illustration of a gold nanoantenna used for four-wave mixing, with an SEM image of the fabricated sample array shown in the inset. (d, e) Extinction cross-section (ECS) of single gold nanoantenna for different incident polarizations, as indicated by the inset. The measured spectra are shown by broken red lines with the simulated spectra shown in solid blue. The pump and seed wavelengths of interest are indicated by the colored vertical bars.

Characterizing the spectral correlations of signal/idler photons is a difficult task for SPDC and SFWM alike, as the generated photon count rates are low. The task is even harder considering the fluorescence background of most materials. Quantum state tomography (QST) is an established method for reconstructing the density matrix describing a two-level photonic system via coincidence measurements,^18^ but this method requires single-photon detectors and long integration times while suffering from a poor signal-to-noise ratio.^19–21^ Stimulated emission tomography (SET), a method proposed by Liscidini and Sipe, greatly simplifies the task and avoids the use of single photon detectors by establishing a relationship between a spontaneous parametric process and its stimulated analog:^22^

$$\frac{\langle n_{\omega_{s}}\rangle_{A_{\omega_{i}}}}{\left\langle n_{\omega_{s}}n_{\omega_{i}}\right\rangle }\approx |A_{\omega_{i}}{|}^{2}$$ 1

here, $\langle n_{\omega_{s}}\rangle_{A_{\omega_{i}}}$ is the average number of signal photons with frequency ω_s_ stimulated by an idler seed with frequency ω_i_, ⟨*n*_ω_s__ *n*_ω_i__⟩ is the average number of photon pairs that would be generated in the spontaneous process, and |*A*_ω_i__|^2^ is the average photon number within the coherence time of the seed.^19,20,23,24^ This enables an indirect characterization of SPDC or SFWM by performing a classical measurement of either difference frequency generation (DFG) or stimulated four-wave mixing (FWM), respectively, which are brighter processes that can be measured by a spectrometer or low-noise photodiode. Furthermore, it is possible to recover the joint spectral density (JSD) of the spontaneous process by scanning the seed beam across the idler wavelengths. The JSD obtained in this manner is of higher resolution than that of spectrally resolved coincidence measurements and is limited only by the line width of the seed beam.^19,20,23,24^

Previous experimental demonstrations of SET have been applied to characterize nonlinear crystals,^24–26^ photonic waveguides,^20,27^ or optical fibers.^28^ SPDC generation rates have been predicted from sum frequency and second harmonic generation in nanophotonic structures,^12,29^ however, these are not stimulated analogues of SPDC and their predictive power is based on the reciprocity principle. Here, we expand on previous work^30^ to demonstrate the SET of a subwavelength parametric light source. SET is especially relevant for the rapidly growing field of nanoantennas and metasurfaces, as the reduced nonlinear conversion efficiencies and greater precision required make coincidence measurements prohibitively difficult. The validation of the SET for these materials may facilitate the characterization and development of nanoscale sources of correlated photon pairs in the future. Our investigation reveals that, with an optimized geometry, the intrinsic SFWM generation rate could reach the order of 10^3^ photon pairs per second per mW squared per antenna. For the unoptimized system considered in this paper, we observe that the extrinsic generation rate is approximately 1 photon pair per second per mW squared per antenna. When scaled up into a metasurface array, photon pair production rates could be sufficient for applications, such as quantum communication, quantum sensing, and integrated photonic circuits.

## Results and Discussion

### Sample and Setup

The sample used in this experiment is a square array of multiparticle gold nanoantennas, each composed of a bar and two disks (Figure 1c). This antenna structure has proven to be extremely effective in previous studies of second- and third-order frequency mixing,^31^ but here we focus on the FWM of individual antennas to evaluate SFWM efficiency. The linear extinction spectra of the antenna arrays were measured with a Fourier transform infrared microscope and converted to extinction cross-section spectra (Figure 1d,e). The antennas support multiple resonances at wavelengths of 1500 and 750 nm and were designed for optimal second-harmonic generation (SHG) emission into the far field.^31^ For the purposes of the FWM measurements in this experiment, however, we take advantage of the field enhancements near 840 and 1500 nm. As can be seen in Figure 1d,e, the chosen excitation wavelength is close to the optimal coupling conditions to the plasmonic antenna. The disk resonance near 750 nm also extends to our chosen pump wavelength in this work, at 840 nm. Meanwhile, the bar resonance near 1500 nm can be accessed either by a seed laser for FWM up-conversion or by radiating the FWM down-conversion. Although the structure is not resonant at 583 nm for either seeding FWM down-conversion or radiating FWM up-conversion, the modest scattering available is sufficient.

A schematic of the experimental setup is shown in Figure 2a. The stimulated FWM measurements involve two input beams: a strong pump beam and a weak seed beam. The pump beam is provided by a 140 fs, 80 MHz Ti:sapphire laser (Coherent Chameleon Ultra II) operating at 840 nm. The same laser further pumps an optical parametric oscillator (APE Compact OPO), which provides a tunable seed beam. In up-conversion measurements, the OPO is tuned around a central wavelength of 1500 nm, which generates a FWM signal near 583 nm when combined with the pump. In down-conversion measurements, the output of the OPO further pumps an SHG frequency converter to bring the seed wavelength into the visible range. The seed beam in this case is tuned around a central wavelength of 583 nm and generates an FWM signal near 1500 nm when combined with the same pump. The pump and seed beams are collinear and focused through the same high numerical aperture (NA = 0.85) objective lens to provide focused beam illumination of the sample at normal incidence. The pump and seed beams are near-diffraction-limited in order to excite a single nanoparticle. In reality, the surrounding particles are partially excited. The 840 nm pump beam is always polarized perpendicular to the bar particle, and the seed beam (1500 nm up-conversion, 583 nm down-conversion) is polarized parallel to the bar. The FWM signal is collected in transmission by a second objective lens and directed to a spectrometer for analysis.

**Figure 2:**
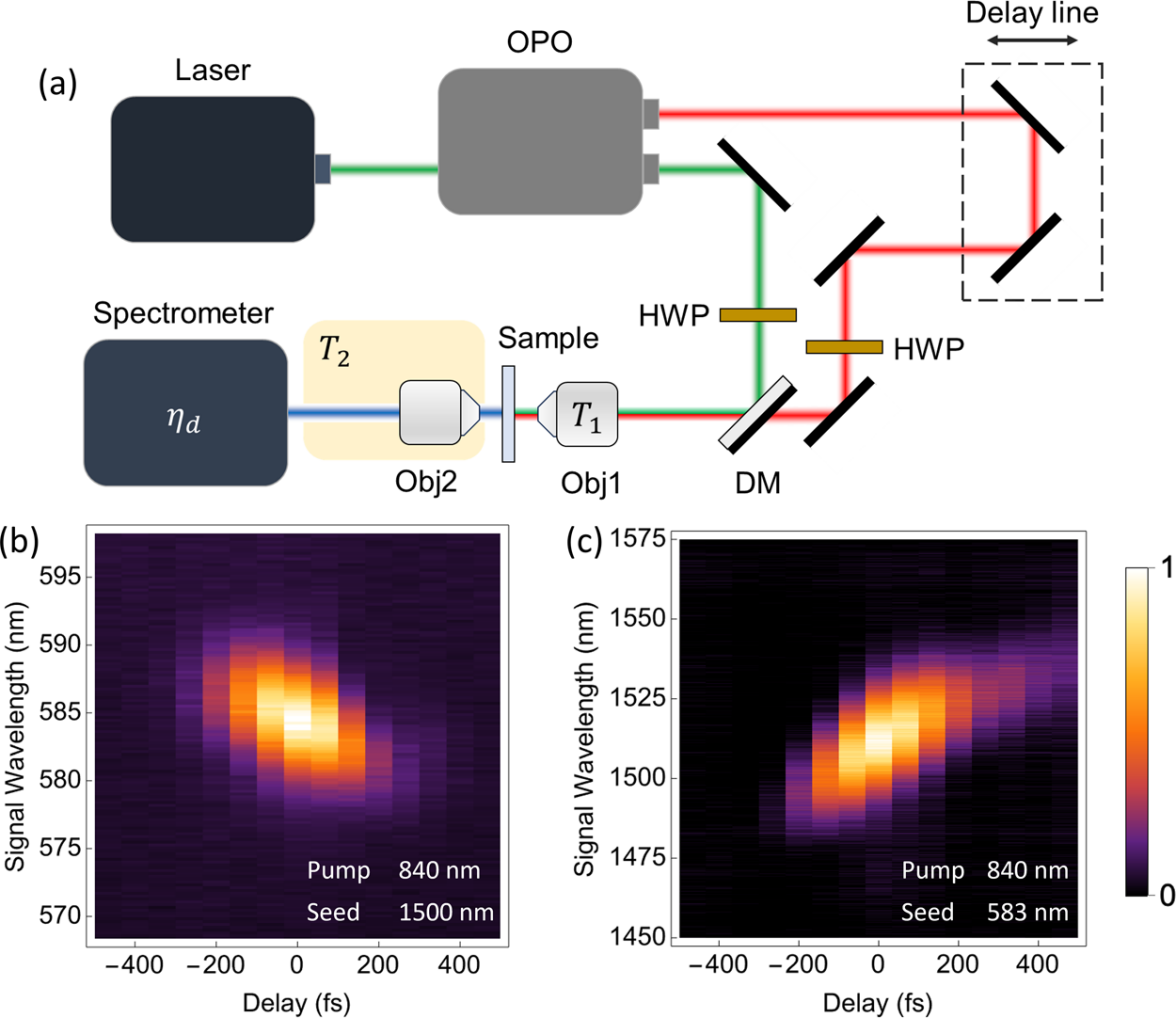
Schematic of experimental setup for four-wave mixing and spectrograms of intensity vs wavelength/delay. (a) Pulsed laser provides the pump beam while simultaneously pumping an optical parametric oscillator (OPO) which outputs the seed beam. A delay line in the seed beam path allows for temporal overlap of both pulses, while a half-wave plate (HWP) controls the polarization in each beam. The two beams are overlapped on a dichroic mirror (DM) and focused collinearly by a 60×/0.95NA objective (Obj1) onto the sample. The generated FWM light is collected by a 40×/0.6NA objective (Obj2) and directed to a spectrometer for analysis. The focusing objective has a different transmissivity for pump (*T*_1,p_) and seed (*T*_1,s_) wavelengths, and the total transmissivity of the FWM signal between the sample and the spectrometer is calibrated separately as *T*_2_, as is the detection efficiency of the spectrometer at the FWM wavelength, η_d_. Spectrograms of intensity vs wavelength/delay for (b) up-conversion and (c) down-conversion FWM. The wavelengths of the pump and seed beams are labeled in each figure with the intensity normalized by the respective maximum value.

### Observation of Four-Wave Mixing

As we are working with ultrafast pulsed sources for both the pump and seed beams, it is crucial that the pulse trains of the two beams are temporally overlapped to observe the frequency mixing. To this end, a piezo-controlled delay stage in one of the beam paths allows observation of the FWM at the correct time delay. Figure 2b,c shows FWM spectrograms of the nanoantenna sample as a function of the time delay between the pump and seed pulses. The FWM signal only appears for time delays consistent with the 140 fs pulse duration of the pump, which shows that the signal is indeed the result of the frequency mixing of the two beams. The signal beam of this system is slightly chirped, which is attributed to the dispersive optics in the beam paths.^32^ Reliable SET measurements require careful calibration of the pump and seed pulse delay offsets, which vary dramatically across the tuning range. This is shown in Figure 3.

**Figure 3:**
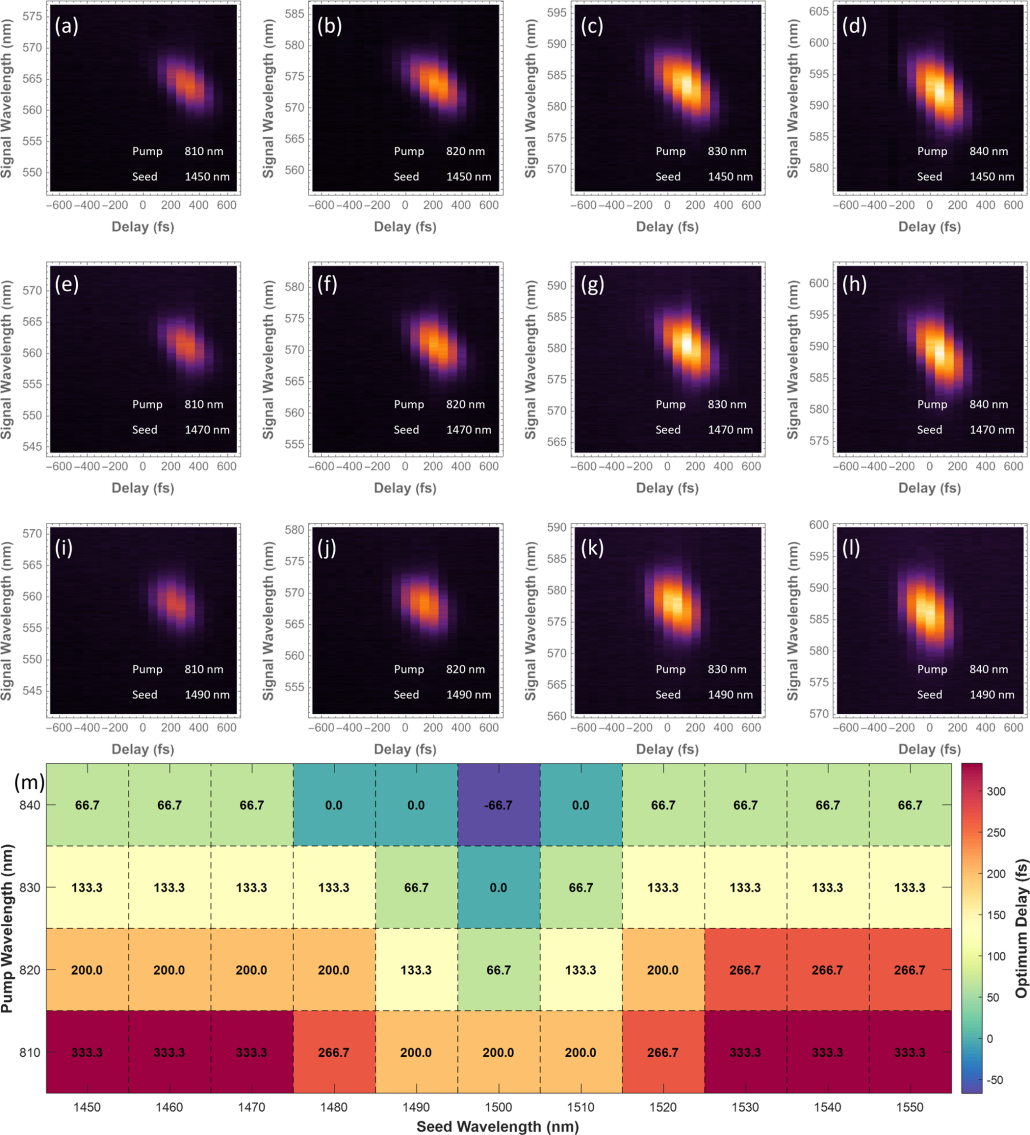
Pulse calibration and spectrograms of intensity vs wavelength/delay for up-conversion FWM. (a–l) Representative spectrograms of intensity vs wavelength/delay for up-conversion FWM. The pump and seed wavelengths are labeled in each figure with the intensity normalized by the respective maximum value. The color bar is identical with the one used in Figure 2. (m) Optimum delay for different combinations of pump wavelengths (810–840 nm) and seed wavelengths (1450–1550 nm).

The observation of down-conversion signals from the gold antennas is a key development in this work, where previously these antennas were measured in FWM up-conversion^31,32^ with a low-noise 2D-array silicon detector. The down-conversion measurements in this work were considerably more challenging given the weak near-infrared emission near 1500 nm, the low field enhancement of the seed fields near 583 nm, and the much more noisy InGaAs detector technology. The observation of both up- and down-conversion FWM signals also shows the unusual situation where visible- and infrared-photon pairs are generated in the same antennas. Typically, SFWM is observed over narrow spectral ranges due to phase-matching restrictions.

### JSD Characterization

Having observed the FWM signals for both up- and down-conversion, we constructed JSD maps of SFWM photon pairs by scanning the wavelength of the seed beam. The wavelength was tuned via the OPO, and great care was taken to select the delay corresponding to the optimum FWM signal at each tuning value. We found that the temporal pulse overlap changed significantly with wavelength tuning. Figure 4 shows the JSD for up- and down-conversion SFWM. The signal wavelength shifts linearly with the seed wavelength, which corresponds to the energy conservation of FWM and further validates the occurrence of frequency mixing. Notably, the JSD is extremely broad, showing the lack of phase matching of parametric fluorescence from a single optical antenna.

**Figure 4:**
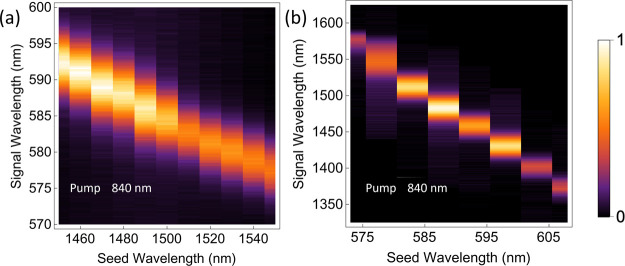
JSD characterization. JSD maps for (a) up-conversion and (b) down-conversion FWM. The wavelength of the pump beam is 840 nm in both figures, and the data are normalized by the respective maximum value. Although the resonance extends beyond the seed wavelength range, a resonance peak near 1470 and 590 nm is visible in (a) and (b) respectively.

The FWM appears to be optimal near 1470 nm for upconversion (Figure 4a) and 590 nm for downconversion (Figure 4b), which can be attributed to the antenna resonance of the bar. The antenna resonance is much broader than the tuning range of the various beams of our laser system, hence the JSD changes relatively little in these maps. It is not possible in this case to scan much further the seed beam wavelengths due to the fixed pump wavelength and an instability of the OPO near 1425 nm. It is possible that the variations over the FWM JSD here are due to alignment drifts during measurements. This should be considered as a possibility given the greater width of the apparent resonances compared to the wavelength scanning range of the seed beam. Regardless of the nature of the signal variations in this experiment, we show that this method can be used to identify narrow resonant features in frequency mixing signals. The shape of the JSD trace in Figure 4 demonstrates that the broadband photon in an SFWM experiment with suitable filtering would exhibit significant spectral correlations. For applications where heralded pure photons are desired, a far narrower resonance, such as a surface lattice resonance^33^ or a bound state in the continuum, would be needed to reach the corresponding circular shape of the JSD.^34^ Note that the JSD is not only useful for photon-pair production but also reveals key information about the classical frequency mixing process itself, such as the relative efficiencies for various wavelength combinations.

### Spontaneous Photon-Pair Generation Rate

The ease with which SET produces JSD maps of parametric processes is already a powerful application, but SET also enables the estimation of the spontaneous photon-pair generation rate from a measurement of the analogous stimulated process. According to eq 1, in order to arrive at the SFWM pair generation rate, we first need to evaluate from our stimulated FWM measurements: (1) the rate of incoming stimulating photons and (2) the rate of generated FWM photons.

The rate of stimulating photons, *ṅ*_s_, is evaluated from the stimulating seed beam power coupled to the antenna, *P̅*_s_,

$${\overset{\cdot }{n}}_{s}=\frac{{\overset{-}{P}}_{s}}{\hbar \omega_{s}}=\frac{P_{s}T_{1,s}\sigma_{s}}{\hbar \omega_{s}A_{s}}$$ 2

where *P*_s_ is the seed beam power at the input of the focusing objective, *T*_1,s_ is the transmission of the objective at the seed wavelength, σ_s_ is the antenna cross section at the seed wavelength, ω_s_ is the central frequency of the seed beam, and *A*_s_ is the area of the seed beam at the focus. In this report, all beam areas correspond to beam radii where the field drops to e^–2^.

To calculate the rate of generated FWM photons, *ṅ*_FWM_, the detector count rate, *ṅ*_cnt_, is divided by the total transmittance of the optical system between the particle and detector at the FWM wavelength, *T*_2_, and the detector efficiency at the FWM wavelength, η_d_,

$${\overset{\cdot }{n}}_{\mathrm{FWM}}=\frac{{\overset{\cdot }{n}}_{\mathrm{cnt}}}{T_{2}\eta_{d}}$$ 3

We can now use eq 1 to relate the spontaneous four-wave mixing rate to the stimulated four-wave mixing rate,

$${ṅ}_{\mathrm{SFWM}}=\frac{{ṅ}_{\mathrm{FWM}}}{{ṅ}_{s}}\Omega$$ 4

where we have multiplied the SET ratio by the number of pulses per second, Ω, to recognize that FWM occurs during each pulse, with the pulse duration setting the coherent interaction time.

Since the SFWM generation rate scales quadratically with the pump power coupled to the antenna, *ṅ*_SFWM_ = *R*_SFWM_^(int)^ *P̅*_p_^2^, we define the intrinsic power-independent generation rate per particle,

$$R_{\mathrm{SFWM}}^{\left(\mathrm{int}\right)}=\frac{{ṅ}_{\mathrm{SFWM}}}{N{\overset{®}{P}}_{p}^{2}}=\left(\frac{\hbar \omega_{s}}{P_{p}^{2}P_{s}}\right)\left(\frac{{ṅ}_{\mathrm{cnt}}}{NT_{1,p}^{2}T_{1,s}T_{2}\eta_{d}}\right)\left(\frac{A_{p}^{2}A_{s}}{\sigma_{p}^{2}\sigma_{s}}\right)\Omega$$ 5

Here, we have substituted eqs 2–4 as well as *P̅*_p_ = *P*_p_ *T*_1,p_σ_p_/*A*_p_, which accounts for the pump power at the input of the objective, *P*_p_, the objective transmission at the pump wavelength, *T*_1,p_, the antenna’s cross section at the pump wavelength, σ_p_, and the pump beam area *A*_p_. The rate is also normalized against the number of particles, *N*, contributing to the FWM. The intrinsic rate normalizes away both the pump power scaling as well as the inefficiency of coupling the pump to the antenna, η_p_ = σ_p_/*A*_p_ ≈ 2%, providing an upper estimate for the photon generation rate under ideal experimental conditions. As this is larger than the power-dependent count rate experimentally achieved, *ṅ*_SFWM_, we also define a power-independent extrinsic generation rate, *R*_SFWM_^(ext)^ = η_p_^2^ *R*_SFWM_^(int)^, which accounts for the inefficient coupling of the pump to the antenna in our experiment.

The transmissions (*T*_1,p_, *T*_1,s_, *T*_2_), beam areas (*A*_p_, *A*_s_), and detector efficiency (η_d_) were measured and calibrated in the experimental setup for both up- and down-conversion wavelengths (Figure 2a), and the antenna cross sections at pump and seed wavelengths (σ_p_, σ_s_) were estimated from extinction spectra measured by FTIR spectroscopy (Figure 1d,e). All calibration data are listed in Table 1.

**Table 1:** Experimental Parameters for Computing SFWM Photon-Pair Generation Rates

	up-conversion	down-conversion
*ṅ*_cnt_	35 200 s^–1^	4610 s^–1^
*P*_p_	2 ± 0.03 mW	3 ± 0.03 mW
*P*_s_	2 ± 0.03 mW	1 ± 0.02 mW
*T*_1,p_	0.598 ± 0.030	0.598 ± 0.030
*T*_1,s_	0.317 ± 0.013	0.887 ± 0.034
*T*_2_	0.797 ± 0.032	0.263 ± 0.013
η_d_	0.00687 ± 0.0007	0.00110 ± 0.0001
*A*_p_	6.79 μm^2^	6.79 μm^2^
*A*_s_	7.80 μm^2^	1.18 μm^2^
σ_p_	0.133 μm^2^	0.133 μm^2^
σ_s_	0.420 μm^2^	0.0875 μm^2^
Ω	80 MHz	80 MHz
*N*	3.02	3.02

We must finally account for the number of particles, *N*, contributing to the FWM, as the pump beam partially overlaps with neighboring particles in the array. We thus compute the overlap of the squared pump field distribution that produces FWM with the antenna positions in our sample. The number of antennas contributing to FWM, *N* = ∑_**R**_e^–4π**R**·**R**/*A*_p_^ ≈ 3.02, where **R** is a square lattice vector with a length of 750 nm.

We are now equipped to estimate the SFWM generation rate of our nanoantennas from the stimulated FWM data. We first consider the case of up-conversion FWM with λ_p_ ≈ 840 nm, λ_s_ ≈ 1500 nm, and λ_FWM_ ≈ 583 nm. Using eqs 2 and 3, we calculate *ṅ*_s_ ≈ 2.58 × 10^14^ photons per second and *ṅ*_FWM_ ≈ 6.43 × 10^6^ photons per second, respectively. Applying eq 5 yields *R*_SFWM_^(int)^ ≈ 1198 photon pairs per second per mW squared per antenna (p.a.). In the down-conversion case, the seed and signal wavelengths are interchanged such that λ_p_ ≈ 840 nm, λ_s_ ≈ 583 nm, and λ_FWM_ ≈ 1500 nm. Applying the same methodology as above yields a spontaneous pair generation rate of *R*_SFWM_^(int)^ ≈ 1762 photon pairs per second per mW squared p.a·. Table 2 summarizes the above results.

**Table 2:** SFWM Photon-Pair Generation Rates

	up-conversion	down-conversion
*ṅ*_s_	2.58 × 10^14^ s^–1^	1.93 × 10^14^ s^–1^
*ṅ*_FWM_	6.43 × 10^6^ s^–1^	1.59 × 10^7^ s^–1^
*ṅ*_SFWM_	2.00 s^–1^	6.61 s^–1^
*R*_SFWM_^(int)^	1198 s^–1^ mW^–2^ p.a.	1762 s^–1^ mW^–2^ p.a.
*R*_SFWM_^(ext)^	0.479 s^–1^ mW^–2^ p.a.	0.705 s^–1^ mW^–2^ p.a.

There are several observations to note here. First, we expect the up and down conversion rates to be of similar values due to the symmetry of the process. The values differ by less than 50%, which is encouraging, while we acknowledge sources of error, including the extensive calibration of detection efficiency and system transmission at the various wavelengths. The normalization procedure developed for antennas here has nonetheless worked fairly well, compensating for the different seeding conditions: in the case of up-conversion, the bar resonance aligns with the seed wavelength, while in down-conversion, the seed tuning to a resonance of the antenna is only partial. Second, for reasons discussed above, the nanoantenna samples in this work were not optimized for FWM emission, and the ranges of wavelengths we work with are constrained by our laser and the OPO system. Using multiresonant antennas, potentially at different wavelength combinations, may increase the pair generation rate. Notably, the difference between the internal and external pair generation efficiencies suggests a large scope for improvement if pump light can be coupled to the structures more effectively. Finally, we have operated with relatively low average pump powers (2 mW for up-conversion and 3 mW for down-conversion) due to the damage threshold of our gold nanoantennas at about 5 mW in a diffraction-limited spot. The pair generation rate would be greatly increased using a metasurface design with wide area illumination of nanoantennas with higher average pump power. Indeed, pair generation rates are quoted in units of inverse power squared and per antenna (p.a.). Expanding the pump beam to excite a metasurface of uncoupled antennas, while maintaining the local intensity, would increase the pair generation rates linearly with the number of antennas excited. Metasurfaces of coupled antennas could also be explored, where the coupling would establish energy-momentum dispersion, affecting the joint spectral density and potentially further enhancing pair generation rates through collective modes.

## Conclusions

In conclusion, we have studied the FWM from subwavelength plasmonic nanoantennas that support a single resonance at either the seed or signal wavelength in both up and down conversion. We have applied SET to construct joint spectral density maps, which reveal the spectral correlations of signal and idler photons that would be generated by SFWM in the antenna. We have also used SET to infer the intrinsic and extrinsic SFWM photon-pair generation rates of our nanoantennas for a particular configuration of wavelengths. We estimate these two values to be ∼10^3^ and ∼1 photon pairs per second per mW squared and per antenna. As this is a fairly conservative estimate, we expect that the spontaneous pair generation rate can be much higher by optimizing the nanoantenna design and moving to the wide-area illumination of a derivative metasurface.

We believe SFWM to be a promising alternative to SPDC for generating photon pairs in metasurfaces, as it is a third-order nonlinear process open to all materials, scales quadratically with pump power, and produces broader bandwidth signal/idler photons. To realize photon pair sources by this route, low-loss dielectric metasurfaces will need to be explored to avoid background fluorescence inherent in absorbing materials. We also find SET to be a convenient method for characterizing the spectral properties of photon pairs generated via spontaneous parametric processes, as it avoids background noise. The evaluation of pair-generation rates in plasmonic antennas would not have been possible otherwise. These findings will support ongoing efforts to design more efficient metasurfaces and nanoscale sources of correlated photon pairs.

## Methods

### Sample Fabrication

The gold nanoantenna arrays were fabricated on silica glass by using standard electron beam lithography (Raith eLINE Plus). The substrate was baked at 180 °C for 5 min after being coated with a positive-tone PMMA resist (950 K A4). The sample was baked at 90 °C for 1 min after coating with a conductive polymer (Espacer 300Z, Showa Denko). The nanostructures were then defined by electron beam exposure, followed by a development procedure. The gold layer was deposited at 2Å/s, followed by a final lift-off process.

### Linear Electromagnetic Simulations

To investigate the linear optical response of the nanoantennas, the optical spectra and electric field distributions were calculated using the finite-difference time-domain (FDTD) method (Lumerical FDTD). Simulations were conducted on a nanoantenna modeled as a gold (Au) bar with a cross-sectional geometry of 340 × 80 nm and Au disks with a diameter of 160 nm, lying on a silicon dioxide (SiO_2_) substrate. The dielectric functions of Au and SiO_2_ were adopted from the data of Johnson and Christy^35^ and Palik,^36^ respectively. Perfectly matched layers (PMLs) were applied at all boundaries to absorb incident light with minimal reflections, and a total-field scattered-field (TFSF) source was employed to efficiently model the polarized plane-wave excitation. The polarized incident wave was applied, normal to the antenna configurations. The mesh quality was validated through a convergence test to ensure the accuracy. The absorption and scattering cross sections were calculated by using the cross-section analysis groups, which measure the net power flowing in and out of defined regions around the structures. The extinction cross-section was determined as the sum of the absorption and scattering cross-sections.
